# Extracellular Vesicle-Mediated Delivery of Curcumin Suppresses Tumor Progression in Murine Oral Squamous Cell Carcinoma

**DOI:** 10.3390/cancers18101586

**Published:** 2026-05-13

**Authors:** Nils Ludwig, Carolin Feldmann, Silvia Spoerl, Saigopalakrishna S. Yerneni

**Affiliations:** 1 Department of Internal Medicine 5-Haematology and Oncology, University Hospital Erlangen, Friedrich-Alexander-University Erlangen-Nürnberg (FAU), 91054 Erlangen, Germany; 2 Department of Pathology, University of Pittsburgh School of Medicine, Pittsburgh, PA 15213, USA; 3 UPMC Hillman Cancer Center, Pittsburgh, PA 15213, USA; 4 Department of Medical Oncology, Heidelberg University Hospital, Medical Faculty Heidelberg, Heidelberg University, 69120 Heidelberg, Germany; 5 Department of Laboratory Medicine and Pathology, Mayo Clinic, 200 First Street SW, Rochester, MN 55905, USA

**Keywords:** oral squamous cell carcinoma, extracellular vesicles, curcumin, nanomedicine, drug delivery, 4-NQO mouse model

## Abstract

**Rationale:** Oral squamous cell carcinoma (OSCC) carries a poor prognosis despite advances in multimodal therapy. Nanomedicine represents a compelling strategy to enhance targeted drug delivery and improve therapeutic outcomes. Here, we investigated sEV-mediated delivery of curcumin as a novel therapeutic approach for OSCC. **Methods:** Small extracellular vesicles (sEVs) were isolated from Jurkat cells by size-exclusion chromatography and loaded with curcumin via sonication to generate JCsEV. Functional effects were assessed in vitro using wound healing, transwell invasion, and metabolic activity assays across multiple cancer cell lines. Therapeutic efficacy in vivo was evaluated in the 4-nitroquinoline 1-oxide (4-NQO) immunocompetent murine model of oral carcinogenesis. Female C57BL/6J mice received intraperitoneal treatment for four weeks with PBS, free curcumin, unloaded JsEV, or JCsEV. Tumor number, tumor burden, and body weight changes were assessed at the experimental endpoint. **Results:** In vitro, JCsEV significantly inhibited tumor cell migration, invasion, and metabolic activity compared with controls (*p* < 0.05). In vivo, treatment with JCsEV significantly reduced tumor number and tumor burden in the 4-NQO model (*p* < 0.01). In addition, body weight loss was reduced in JCsEV-treated mice compared with controls. **Conclusion:** sEV-mediated delivery of curcumin effectively suppresses tumor progression in experimental OSCC. These findings establish proof-of-concept for sEV-based nanomedicine as a therapeutic strategy for OSCC and provide a compelling rationale for further translational investigation of sEVs as drug delivery platforms.

## Simple Summary

Oral squamous cell carcinoma (OSCC) remains a therapeutic challenge, and novel treatment strategies are critically needed. Curcumin is a naturally occurring compound with well-documented anti-cancer properties; however, its clinical application is constrained by poor aqueous solubility and rapid systemic elimination. In this study, we harnessed small extracellular vesicles (sEVs) as carriers for targeted curcumin delivery. Curcumin-loaded sEVs (JCsEV) suppressed tumor cell metabolic activity, migration, and invasion in vitro. In an immunocompetent murine model of oral carcinogenesis, JCsEV significantly reduced tumor number and tumor burden compared with controls and showed a more pronounced therapeutic effect than free curcumin. These findings support the concept that sEV-based delivery enhances the therapeutic potential of curcumin and represents a promising translational approach for OSCC.

## 1. Introduction

Oral squamous cell carcinoma (OSCC) is the most prevalent malignancy of the oral cavity and represents a major global health burden [1]. Despite intensive research and the development of new therapeutic strategies over recent decades, clinical outcomes have improved only marginally. Consequently, 5-year survival rates have remained largely unchanged, with absolute survival rates of approximately 55% [1,2].

Small extracellular vesicles (sEVs) are a subpopulation of extracellular vesicles ranging from approximately 30 to 150 nm in diameter, which are constitutively secreted by virtually all cell types. Their biogenesis, molecular composition, and functional properties are determined by the cell of origin and the prevailing physiological or pathological context [3,4]. sEVs contain a complex cargo of proteins, lipids, and various classes of nucleic acids, including mRNA, microRNA and DNA [5,6]. Importantly, their proteomic and nucleic acid profiles largely reflect the cytosolic content and surface composition of their parent cells, enabling them to transfer biologically relevant information [7,8]. Consequently, sEVs function as key mediators of intercellular communication capable of engaging recipient cells through ligand–receptor interactions or direct internalization, thereby modulating intracellular signaling pathways of recipient cells and reprogramming their functionality [9,10,11]. These intrinsic properties render sEVs particularly attractive as nanocarrier systems with therapeutic potential. The abundant surface adhesion molecules of sEVs and their natural role in cell-to-cell communication facilitate the targeted delivery of encapsulated cargo to specific recipient cell populations [12]. Furthermore, the lipid bilayer membrane shields encapsulated molecules from enzymatic degradation, prolongs systemic stability, and enables efficient—and potentially cell-type-selective—delivery [13].

Curcumin is a bioactive polyphenolic compound extracted from the rhizome of *Curcuma longa* and possesses well-established anti-inflammatory and antineoplastic properties [14]. It concurrently modulates multiple oncogenic signaling pathways involved in tumor cell metabolic activity, invasion, and inflammation [14,15]. Despite this promising pharmacological profile, the clinical translation of curcumin is hindered by poor aqueous solubility, limited oral bioavailability, and rapid systemic clearance [16]. Nanocarrier-based delivery strategies have therefore been pursued to circumvent these pharmacokinetic limitations and enhance curcumin’s therapeutic efficacy [13,15]. Loading curcumin into sEVs represents a particularly promising approach, as this strategy may simultaneously improve drug stability, bioavailability, and tumor-targeted delivery [13]. However, the therapeutic potential of sEV-mediated curcumin delivery for OSCC, particularly in immunocompetent in vivo models, has not been systematically investigated.

To address this gap, we evaluated curcumin-loaded sEVs as a novel therapeutic strategy in OSCC using functional in vitro assays and the 4-nitroquinoline 1-oxide (4-NQO) murine model of oral carcinogenesis.

## 2. Materials and Methods

### 2.1. Cell Lines and Culture Conditions

Jurkat cells (ATCC^®^ TIB-152™, ATCC, Manassas, VA, USA), the lung adenocarcinoma cell line A549 (ATCC, Manassas, VA, USA), the HPV-negative OSCC cell line PCI-30 (established by Dr. Theresa Whiteside, UPMC Hillman Cancer Center, Pittsburgh, PA, USA), and the HPV-positive laryngeal squamous cell carcinoma cell line UMSCC90 (established by Dr. Thomas Carey, University of Michigan, Ann Arbor, MI, USA) were used in this study. Jurkat cells were cultured in RPMI-1640 supplemented with 10% sEV-depleted, heat-inactivated fetal bovine serum (FBS) and 1% penicillin/streptomycin at 37 °C in 5% CO_2_. Prior to use, FBS was depleted of endogenous sEVs by ultracentrifugation at 100,000× *g* for 3 h, followed by heat inactivation at 56 °C for 30 min, and filtration through 0.22 µm filters. A549, PCI-30, and UMSCC90 cells were cultured in DMEM supplemented with 10% sEV-depleted, heat-inactivated FBS and 1% penicillin/streptomycin under identical conditions (37 °C, 5% CO_2_).

### 2.2. sEV Isolation

sEVs were isolated from conditioned cell culture supernatants of Jurkat cells by size-exclusion chromatography (SEC), as previously described [17]. Conditioned supernatants were collected after 72 h and clarified by sequential centrifugation at 2000× *g* (10 min, room temperature) and 10,000× *g* (30 min, 4 °C), followed by 0.22 µm filtration (Merck KGaA, Darmstadt, Germany). Twenty-five milliliter aliquots were concentrated using Vivacell 100 units (MWCO 10,000 kDa; Sartorius Corp, Bohemia, NY, USA) at 2000× *g*. Concentrated supernatant (1 mL) was loaded onto a 1.5 cm × 12 cm Sepharose 2B column (Sigma–Aldrich, St. Louis, MO, USA; Econo-Pac columns, Bio-Rad, Hercules, CA, USA) and eluted with phosphate-buffered saline (PBS). Fraction #4, enriched in morphologically intact, non-aggregated sEVs, was used in all subsequent experiments.

sEV isolation from human plasma was performed as previously described [17,18]. Peripheral venous blood was obtained from 4 patients with OSCC with active disease who were seen in the Otolaryngology Clinic between 2016 and 2019. Samples were obtained from the University of Pittsburgh SPORE bank under Institutional Review Board-approved protocols of the University of Pittsburgh. In addition, blood from healthy volunteers was obtained from 4 consented donors under the same regulatory framework. Blood samples were centrifuged at 1000× *g* for 10 min to separate plasma. Plasma specimens were stored in 1 mL aliquots at −80 °C and thawed immediately prior to sEV isolation. Plasma samples were processed for sEV recovery as previously described [17].

### 2.3. sEV Characterization

sEVs were characterized according to the MISEV2023 guidelines as previously described [17,19,20]. Protein concentrations were determined with BCA assay (Pierce Biotechnology, Rockford, IL, USA). sEV morphology was assessed by transmission electron microscopy (TEM), as previously described [19]. Briefly, sEVs were fixed in 4% glutaraldehyde for 20 min at room temperature, applied to Formvar-coated copper grids, and negatively stained with uranyl acetate. Samples were examined using a Hitachi H-7100 transmission electron microscope (Hitachi High Technologies, Tokyo, Japan) operated at 100 keV. Particle size distribution and concentration were analyzed by nanoparticle tracking analysis (NTA) using a NanoSight LM10 system (NanoSight Ltd., Amesbury, UK) equipped with a 405 nm laser, as previously described [19].

For Western blot analysis, fraction #4 samples were concentrated using 100 kDa Amicon Ultra centrifugal filters (EMD Millipore, Billerica, MA, USA) at 4000× *g* and loaded at 5 µg protein per lane. Proteins were separated by SDS-PAGE and transferred to PVDF membranes, followed by overnight incubation with primary antibodies at 4 °C, as previously described [10,17]. The following primary antibodies were used: TSG101 (1:500; MA1-23296, Thermo Fisher Scientific, Waltham, MA, USA), Grp94 (1:1000; #20292S, Cell Signaling Technology, Waltham, MA, USA), and CD9 (1:1000; MA5-31980, Thermo Fisher Scientific, Waltham, MA, USA). Cropped blot images are presented in the main figures, while full-length, uncropped membranes are provided in Supplementary Figure S1.

### 2.4. Curcumin Loading into sEVs

Sequential loading of albumin and curcumin into sEVs was accomplished by mild sonication, as previously described [19]. In brief, sEVs were first loaded with bovine serum albumin (BSA) at a 1:1 mass ratio (μg albumin:μg sEV protein) by mild sonication. Surface-adsorbed and unencapsulated albumin were removed by acid rinsing followed by SEC purification; albumin loading efficiency was quantified by BCA assay relative to unloaded sEVs. Curcumin was subsequently loaded into albumin-primed sEVs at a 1:1 mass ratio (μg curcumin:μg albumin-sEV) using an identical sonication protocol, followed by SEC purification. Curcumin encapsulation was quantified by high-performance liquid chromatography (HPLC), as previously described [19]. Using this previously validated protocol, HPLC analysis demonstrated a loading efficiency of 0.56 ± 0.01 μg curcumin per 1 μg albumin-EV [19]. This loading strategy enabled efficient curcumin encapsulation without relevant alterations in sEV size distribution or morphology [19].

### 2.5. Functional In Vitro Assays Using sEVs

To characterize the functional activity of curcumin-loaded sEVs, three in vitro assays were performed. Experiments were conducted using A549, PCI-30, and UMSCC90 cells, as described above. For all functional assays, cells were seeded at a density of 5 × 10^5^ cells per well in 6-well plates (Corning Inc., Corning, NY, USA) and allowed to adhere for 4 h. Cells were then pretreated for 72 h with plasma-derived sEVs from OSCC patients (tumor sEV; 10 µg/mL), sEVs from normal donors (ND sEV; 10 µg/mL), or PBS as vehicle control.

#### 2.5.1. Mitochondrial Dehydrogenase Activity Assay

To assess metabolic activity, A549, PCI-30, and UMSCC90 cells were seeded in 96-well flat-bottom plates at a density of 5 × 10^3^ cells per well in 100 µL of complete DMEM and allowed to adhere for 4 h at 37 °C in 5% CO_2_. Indicated sEV treatments (10 µg/mL) were then added to the wells for 48 h. To measure the metabolic health of the cells, the medium was replaced with PBS and 20 µL of CellTiter 96^®^ AQueous One Solution Reagent (MTS; Promega, Madison, WI, USA) was added to each well. Plates were incubated at 37 °C in 5% CO_2_ for 2 h, and absorbance was measured at 490 nm using a microplate spectrophotometer (Synergy H1, Agilent BioTek, Santa Clara, CA, USA). Background absorbance was measured from medium-only wells and subtracted from all values. Metabolic activity was expressed as fold of PBS control. All conditions were performed in triplicate, and experiments were independently repeated at least twice for each cell line.

#### 2.5.2. Wound Healing Assay

Following pretreatment, cells were reseeded into 24-well plates and allowed to adhere for 4 h. Secondary treatment was applied as follows: PBS-pretreated cells received PBS, curcumin (5.6 µg/mL), or unloaded JsEV (10 µg/mL); ND sEV-pretreated cells received PBS only; and tumor sEV-pretreated cells received PBS, JsEV (10 µg/mL), curcumin (5.6 µg/mL), or JCsEV (10 µg/mL). Upon reaching confluence, a linear wound was introduced with a sterile 200 µL pipette tip. Phase-contrast images were acquired immediately after scratching and at 12 h using an Axiovert 25 CFL inverted microscope (5× magnification; Carl Zeiss Microscopy GmbH, Oberkochen, Germany). Cells were fixed in methanol and stained with 0.2% crystal violet (MilliporeSigma, Burlington, MA, USA). Wound closure was quantified with ImageJ (version 1.54q, National Institutes of Health, Bethesda, MD, USA) and expressed as the percentage of wound area recovered relative to baseline. Each experiment was performed in two independent replicates for each cell line.

#### 2.5.3. Transwell Invasion Assay

Invasive capacity was assessed using Matrigel-coated transwell inserts (8 µm pore size; Corning Inc., Corning, NY, USA) in 24-well plates. Following treatment identical to that described for the wound healing assay, cells were serum-starved overnight and seeded into the upper chamber at a density of 5 × 10^4^ cells in DMEM containing 1% FBS. The lower chamber contained DMEM supplemented with 10% FBS as a chemoattractant. After 6 h of incubation at 37 °C and 5% CO_2_, non-invading cells were removed from the upper membrane surface. Invaded cells on the lower surface were fixed in methanol, stained with 0.2% crystal violet, and quantified by light microscopy. Six random fields per insert were analyzed at 20× magnification, and results are expressed as the mean number of invading cells. Experiments were independently performed twice for each cell line.

### 2.6. Murine Oral Carcinogenesis Model

The 4-nitroquinoline 1-oxide (4-NQO)-induced murine oral carcinogenesis model was used as previously described [21]. Female immunocompetent C57BL/6J mice (8 weeks old; Jackson Laboratories, Bar Harbor, ME, USA) were housed under standard specific pathogen-free conditions with ad libitum access to food and water and a 12 h light/dark cycle. Animals were allowed to acclimatize prior to the start of experimental procedures. Mice were monitored regularly for general health status, body weight, food and water intake, and signs of distress. Humane endpoints were predefined according to the approved IACUC protocol and included excessive weight loss, impaired mobility, severe ulceration, or signs of systemic illness.

To induce the development of oral carcinomas, mice were administered 4-NQO (Tokyo Chemical Industry Co., Ltd., Tokyo, Japan) dissolved in propylene glycol (Sigma–Aldrich; stock 4 mg/mL, diluted to 0.1 mg/mL) in drinking water for 16 consecutive weeks, followed by a washout period with regular water. Beginning at week 18, mice were randomized into four treatment groups and received intraperitoneal injections every 72 h through week 22: PBS (100 μL; n = 6), free curcumin (28 μg in 100 μL PBS; n = 7), unloaded JsEV (50 μg in 100 μL PBS; n = 8), or JCsEV (50 μg in 100 μL PBS; n = 6). No formal a priori sample size calculation was performed; group sizes were based on previous experience with the 4-NQO model and comparable preclinical studies. At the experimental endpoint (week 22), oral lesions were enumerated and tumor dimensions (length, width, height) were measured with a digital caliper. Tumor volume (mm^3^) was calculated using an ellipsoid formula, as previously described [22]. No animals were excluded from the analysis unless predefined humane endpoint criteria were met or tissue quality precluded reliable tumor assessment. All available animals were included in the final analysis. Investigators were not blinded during treatment administration; however, tumor enumeration and volumetric assessment were performed blinded to treatment group whenever possible.

### 2.7. Statistical Analysis

All statistical analyses were performed using GraphPad Prism (v10.4.1). Data are presented as mean ± SEM. Between-group differences were evaluated by one-way ANOVA with Student–Newman–Keuls post hoc testing for pairwise comparisons. Statistical significance was defined as *p* < 0.05.

## 3. Results

### 3.1. Characterization of sEVs and Curcumin Loading

**Figure 1:**
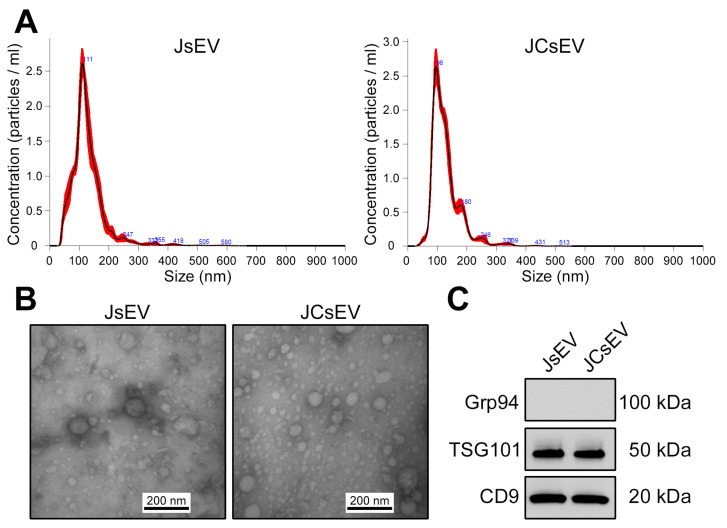
Characterization of JsEV and curcumin-loaded JCsEV. (**A**) Particle size distribution and concentration profiles of JsEV and JCsEV determined by nanoparticle tracking analysis (NTA). Representative measurements demonstrate a predominant vesicle population within the expected size range of sEVs. (**B**) Representative transmission electron microscopy (TEM) images of JsEV and JCsEV showing typical cup-shaped vesicles with heterogeneous size distribution. Scale bars: 200 nm. (**C**) Western blot analysis of JsEV and JCsEV demonstrating the presence of canonical sEV markers (TSG101 and CD9) and the absence of the negative marker GRP94. Each lane was loaded with 5 µg protein. Curcumin loading was performed using a previously HPLC-validated protocol demonstrating a loading efficiency of 0.56 ± 0.01 µg curcumin per 1 µg albumin-EV [19]. The uncropped blots are shown in Supplementary Materials.

JsEV and JCsEV were characterized in accordance with the MISEV2023 guidelines [20]. NTA demonstrated that both preparations exhibited a unimodal size distribution consistent with sEVs, with a mean diameter of 132.9 ± 1.1 nm for JsEV and 126.9 ± 1.8 nm for JCsEV, and particle concentrations of approximately 1.98 × 10^10^ and 1.85 × 10^10^ particles/mL, respectively (Figure 1A). TEM confirmed the presence of intact, spherical vesicles in both preparations, with no evidence of aggregation or morphological disruption following curcumin loading (Figure 1B). Western blot analysis demonstrated expression of the sEV markers TSG101 and CD9 in both JsEV and JCsEV, while the endoplasmic reticulum contaminant marker Grp94 was absent, confirming the purity and identity of the isolated vesicles (Figure 1C). These findings demonstrate that curcumin loading by sonication preserves the structural integrity and molecular identity of the sEV preparations.

Curcumin loading itself was performed according to a previously validated protocol, in which HPLC-based quantification demonstrated a loading efficiency of 0.56 ± 0.01 μg curcumin per 1 μg albumin-EV [19], confirming efficient and reproducible curcumin encapsulation into sEVs. Following isolation and curcumin loading, the biological activity of the sEV preparations was evaluated in functional in vitro assays to assess the effects on tumor cell migration, invasion, and metabolic activity. These experiments were designed to determine whether JCsEV modulate pro-tumorigenic activity and to establish a mechanistic foundation for subsequent in vivo studies using the 4-NQO murine oral carcinogenesis model.

### 3.2. JCsEV Inhibit Tumor Cell Migration

**Figure 2:**
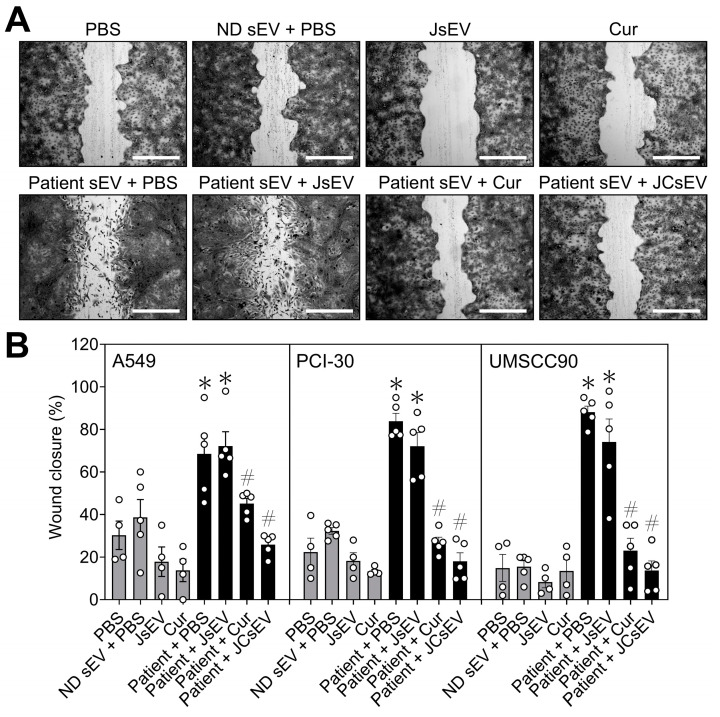
Patient-derived sEVs promote tumor cell migration, which is inhibited by curcumin-loaded JCsEV. (**A**) Representative phase-contrast micrographs of wound healing assays performed with PCI-30 cells 12 h after wound generation. Cells were treated with PBS, ND sEV + PBS, JsEV, curcumin, patient-derived sEV + PBS, patient-derived sEV + JsEV, patient-derived sEV + curcumin, or patient-derived sEV + JCsEV. Scale bar: 100 µm. (**B**) Quantitative analysis of wound closure in A549, PCI-30, and UMSCC90 cells. Data represent the percentage of wound closure 12 h after scratch generation. Bars indicate mean ± SEM from two independent experiments with individual data points shown. * *p* < 0.05 vs. PBS; # *p* < 0.05 vs. Patient + PBS.

Migratory capacity was evaluated by wound healing assay in A549, PCI-30, and UMSCC90 cells. Representative phase-contrast images acquired at 12 h following treatment are shown in Figure 2A. Across all three cell lines, exposure to patient-derived sEVs markedly accelerated wound closure relative to PBS-treated controls and induced pronounced morphological alterations consistent with an EMT-like phenotype, including spindle-shaped morphology and diminished cell–cell contacts. Control conditions (PBS, ND sEV + PBS, JsEV, and curcumin alone) resulted in only modest wound closure with preservation of an intact epithelial monolayer. Treatment of tumor sEV-primed cells with curcumin or JCsEV attenuated migratory activity, with JCsEV producing a particularly pronounced inhibitory effect. Unloaded JsEV did not significantly alter tumor sEV-induced migration (Figure 2A).

Quantitative analysis of wound closure corroborated these qualitative observations (Figure 2B). Patient-derived sEVs significantly enhanced wound closure in all three cell lines compared with PBS controls (*p* < 0.05). Neither ND sEV, JsEV, nor free curcumin alone significantly altered baseline migration. Free curcumin significantly attenuated tumor sEV-induced migration (*p* < 0.05), whereas JCsEV reduced wound closure to near-baseline levels across all three cell lines, resulting in a marked inhibitory effect (*p* < 0.05; Figure 2B).

### 3.3. JCsEV Suppress Tumor Cell Invasion

**Figure 3:**
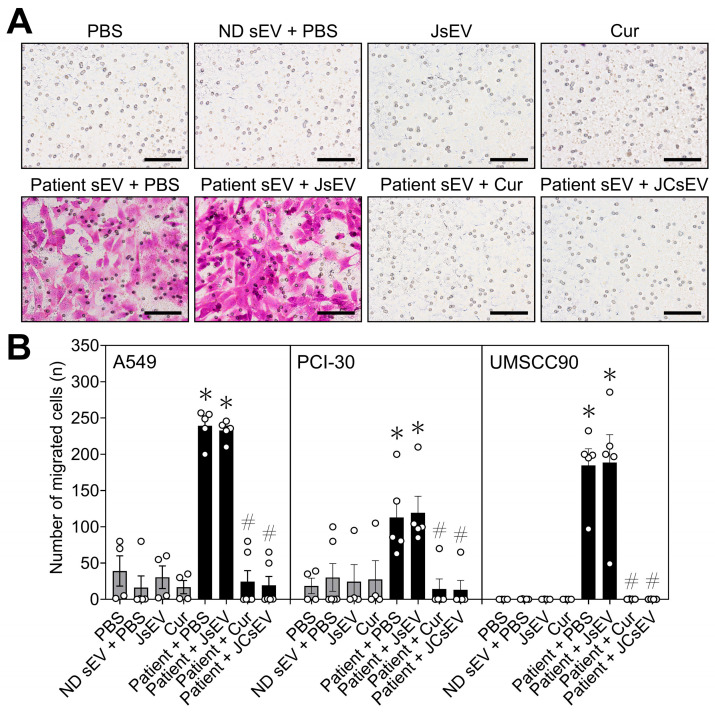
Patient-derived sEVs enhance tumor cell invasion, which is inhibited by curcumin-loaded JCsEV. (**A**) Representative micrographs of transwell invasion assays showing invaded PCI-30 cells after treatment with PBS, ND sEV + PBS, JsEV, curcumin, patient-derived sEV + PBS, patient-derived sEV + JsEV, patient-derived sEV + curcumin, or patient-derived sEV + JCsEV. Invaded cells were fixed and stained with crystal violet. Scale bar: 100 µm. (**B**) Quantitative analysis of invaded cells in A549, PCI-30, and UMSCC90 cell lines. Data represent the number of migrated cells per field. Bars indicate mean ± SEM from two independent experiments with individual data points shown. * *p* < 0.05 vs. PBS; # *p* < 0.05 vs. Patient + PBS.

Tumor cell invasion was evaluated using Matrigel-coated transwell assays. Representative images of invaded cells following treatment with each sEV preparation are shown in Figure 3A. In all three cell lines, patient-derived sEVs significantly increased invasive capacity compared with PBS-treated controls. In contrast, no relevant changes in invasion were observed in the control groups (PBS, ND sEV + PBS, JsEV, or curcumin alone). Quantitative analysis confirmed a significant increase in the number of invaded cells following treatment with patient-derived sEVs in A549, PCI-30, and UMSCC90 cells (*p* < 0.05; Figure 3B). This pro-invasive effect was significantly suppressed by both free curcumin and JCsEV (*p* < 0.05), with JCsEV showing a numerically more pronounced inhibitory effect. Treatment with JsEV alone did not significantly alter invasive behavior (Figure 3B).

### 3.4. JCsEV Reduce Tumor Cell Metabolic Activity

To determine whether the observed effects on migration and invasion were accompanied by changes in tumor cell metabolism, MTS assays were performed in A549, PCI-30, and UMSCC90 cells treated with the respective sEV preparations. Cells were incubated for 24 h in the presence/absence of sEVs, and their metabolic health was quantified using the MTS assay relative to PBS-treated controls.

**Figure 4:**
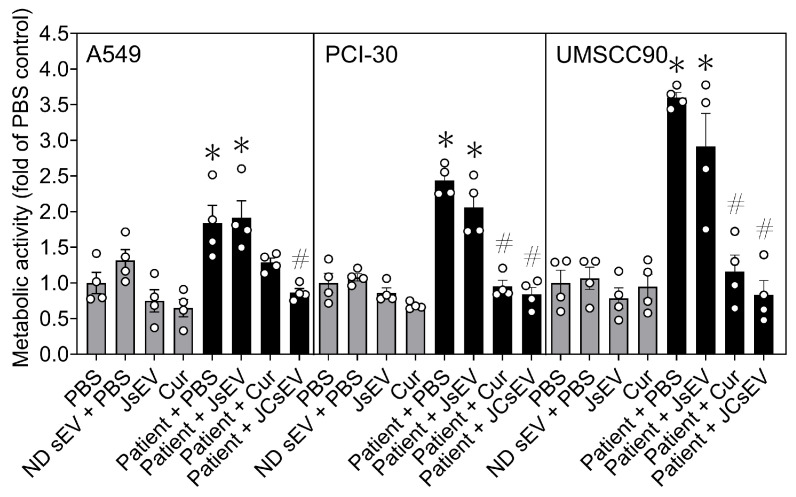
JCsEV reduce tumor cell metabolic activity. Metabolic activity of A549, PCI-30, and UMSCC90 cells was assessed by MTS assay following treatment with PBS, ND sEV + PBS, JsEV, curcumin, patient-derived sEV + PBS, patient-derived sEV + JsEV, patient-derived sEV + curcumin, or patient-derived sEV + JCsEV for 24 h. Data are presented as metabolic activity normalized to PBS-treated controls (fold of PBS control). Bars indicate mean ± SEM from two independent experiments with individual data points shown. * *p* < 0.05 vs. PBS; # *p* < 0.05 vs. Patient + PBS.

Across all three cell lines, treatment with patient-derived sEVs resulted in a significant increase in metabolic activity compared with PBS controls (*p* < 0.05; Figure 4). In contrast, control conditions (ND sEV + PBS, JsEV, and curcumin alone) did not significantly alter baseline metabolic activity. Subsequent treatment of tumor sEV-primed cells with free curcumin significantly reduced metabolic activity compared with patient sEV treatment alone (*p* < 0.05), although levels remained above baseline. Notably, treatment with JCsEV was associated with a more pronounced reduction in metabolic activity, restoring levels close to those observed under control conditions across all three cell lines (*p* < 0.05). Importantly, unloaded JsEV did not significantly inhibit metabolic activity, whereas curcumin-loaded JCsEV significantly reduced patient sEV-induced metabolic activity, indicating that the observed inhibitory effect was primarily associated with curcumin loading rather than unloaded sEV treatment alone. These effects were most prominent in UMSCC90 cells, where patient-derived sEVs induced the strongest increase in metabolic activity and the strongest numerical suppression was observed after JCsEV treatment (Figure 4).

Taken together, these findings indicate that patient-derived sEVs enhance tumor cell metabolic activity, while JCsEV effectively counteract these effects. In conjunction with the observed inhibition of migration and invasion, these data consistently support a suppressive effect of JCsEV on tumor cell aggressiveness. These findings provide a strong mechanistic rationale for subsequent in vivo evaluation in the 4-NQO mouse model.

### 3.5. Curcumin-Loaded JCsEV Suppress Tumor Progression in the 4-NQO Murine Oral Carcinogenesis Model

**Figure 5:**
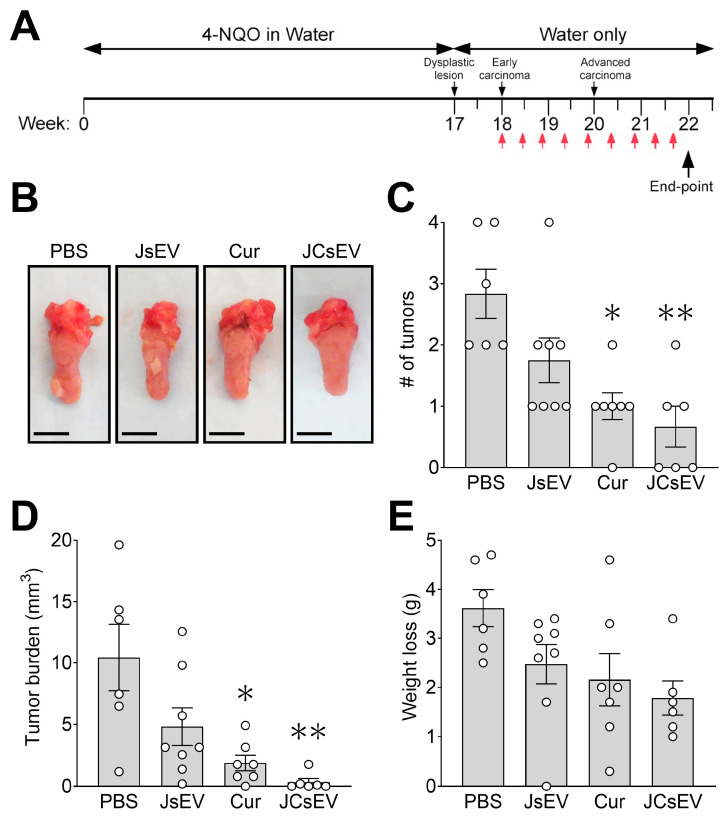
Curcumin-loaded JCsEV reduce tumor burden in the 4-NQO murine oral carcinogenesis model. (**A**) Schematic overview of the experimental design. Mice were exposed to 4-NQO in drinking water for 16 weeks to induce oral carcinogenesis, followed by administration of regular water. From week 18 to week 22, mice received intraperitoneal treatment with PBS, JsEV, curcumin, or curcumin-loaded JCsEV at 72 h intervals. The experimental endpoint was reached at week 22. Red arrows indicate the time points of intraperitoneal treatment administration. (**B**) Representative macroscopic images of resected tongues with visible tumors obtained at the experimental endpoint after treatment with PBS, JsEV, curcumin, or curcumin-loaded JCsEV. Scale bar: 5 mm. (**C**) Number of tumors per mouse at the experimental endpoint following treatment with PBS, JsEV, curcumin, or curcumin-loaded JCsEV. (**D**) Quantitative analysis of tumor burden expressed as tumor volume (mm^3^) per mouse at the experimental endpoint. (**E**) Body weight loss (g) of mice during the experimental period under the indicated treatment conditions. Bars represent mean ± SEM with individual data points shown. * *p* < 0.05 vs. PBS; ** *p* < 0.01 vs. PBS.

To assess the in vivo therapeutic efficacy of JCsEV, mice in the 4-NQO oral carcinogenesis model were randomized at week 18 to receive PBS, unloaded JsEV, free curcumin, or JCsEV intraperitoneally every three days for four weeks. Tumor outcome was evaluated at the experimental endpoint (week 22; Figure 5A). Representative macroscopic images of resected tongues demonstrated visibly reduced tumor burden in curcumin- and JCsEV-treated mice relative to PBS controls (Figure 5B), while all individual tongue samples are shown in Supplementary Figure S2. Quantitative analysis revealed a significant reduction in tumor number per mouse in the curcumin group (*p* < 0.05 vs. PBS), with a numerically more pronounced reduction observed in the JCsEV group (*p* < 0.01 vs. PBS), whereas unloaded JsEV produced only a modest, non-significant reduction (Figure 5C). A similar pattern was observed for overall tumor burden. The greatest numerical reduction in total tumor volume was observed in the JCsEV group (*p* < 0.01 vs. PBS), followed by free curcumin (*p* < 0.05 vs. PBS), while JsEV showed no significant effect relative to the PBS control group (Figure 5D). Body weight loss followed a similar pattern, with the greatest weight loss in PBS-treated mice and partial attenuation in the curcumin and JCsEV groups; however, these differences did not achieve statistical significance (Figure 5E).

Collectively, these findings demonstrate that systemic administration of JCsEV significantly suppresses tumor progression in the 4-NQO oral carcinogenesis model and attenuates tumor-associated weight loss, suggesting that sEV-mediated delivery may enhance the therapeutic activity of curcumin in vivo. However, the direct comparison between JCsEV and free curcumin did not reach statistical significance, precluding a definitive conclusion of superior efficacy.

## 4. Discussion

This study demonstrates that sEV-mediated delivery of curcumin exerts significant anti-tumor effects in experimental OSCC. In vitro, patient-derived sEVs potently promoted tumor cell migration, invasion, and an EMT-like phenotypic transition, underscoring their pro-tumorigenic role within the tumor microenvironment (TME). JCsEV effectively counteracted these effects, consistently suppressing migration, invasion, and metabolic activity across multiple cell lines. Critically, these in vitro findings translated into the in vivo setting, where systemic JCsEV administration significantly reduced both tumor number and tumor burden in the immunocompetent 4-NQO oral carcinogenesis model. Notably, JCsEV showed a numerically stronger therapeutic effect than free curcumin, supporting the hypothesis that sEV encapsulation may enhance the biological activity of curcumin in vivo. Collectively, these results provide compelling proof-of-concept that sEV-based nanocarriers can be leveraged for targeted delivery of anti-tumor compounds in OSCC.

Curcumin-loaded sEVs have been explored across a broad range of preclinical disease models and have demonstrated promising therapeutic activity in diverse experimental settings [23,24,25]. In contrast to conventional single-target agents, curcumin exerts pleiotropic effects by simultaneously modulating multiple molecular targets implicated in inflammation, oxidative stress, and tissue regeneration [23,26]. Several studies have highlighted the therapeutic versatility of sEV-mediated curcumin delivery. For example, curcumin-loaded macrophage-derived sEVs have been shown to accelerate diabetic wound healing by attenuating oxidative stress and inflammation while simultaneously promoting angiogenesis and re-epithelialization [27]. Similarly, sEV-encapsulated curcumin improved tissue perfusion and attenuated ischemic necrosis in preclinical skin flap transplantation models [28]. Beneficial effects have also been reported in vascular and neurological disease models, where curcumin-loaded sEVs improved endothelial function, reduced blood–brain barrier permeability, and mitigated neuroinflammatory injury following ischemia–reperfusion [29]. Taken together, these findings demonstrate the broad pathophysiological relevance of sEV-mediated curcumin delivery and provide a compelling rationale for its translation into oncological applications.

In contrast to the expanding literature in non-oncological diseases, evidence supporting the therapeutic application of curcumin-loaded sEVs in cancer remains limited [30]. While curcumin has been extensively studied in preclinical and clinical oncology, only a small number of investigations have explored sEVs as dedicated delivery vehicles for curcumin. In a pioneering study, Aqil et al. demonstrated that curcumin encapsulated in milk-derived sEVs markedly increased oral bioavailability and exhibited enhanced anti-proliferative activity across several cancer cell lines, as well as tumor growth inhibition in vivo without evidence of systemic toxicity [31]. More recently, Saeed et al. reported that curcumin-loaded camel milk-derived sEVs enhanced cytotoxicity in non-small-cell lung cancer models and suppressed key oncogenic signaling nodes, including EGFR and STAT3 [32]. Together, these studies suggest that sEV-based delivery may enhance the anti-tumor activity of curcumin across multiple cancer types. Notably, curcumin has previously demonstrated chemopreventive and anti-tumor effects in the 4-NQO oral carcinogenesis model, including suppression of cancer stem cell-associated signaling pathways [33]. However, whether sEV-based delivery may further enhance the anti-tumor efficacy of curcumin in the context of oral carcinogenesis remains unknown.

Specifically in OSCC, preclinical evidence for anti-tumor effects of curcumin-loaded sEVs is currently absent. Several prior studies have nonetheless investigated curcumin in the context of alternative nanocarrier platforms. For example, polymer-based nanoparticles, including PLGA formulations, have demonstrated cytotoxic and pro-apoptotic activity in vitro, while more recent combinatorial strategies incorporating curcumin with targeted agents or hydrogel-based delivery platforms have demonstrated anti-tumor efficacy in xenograft models [34,35]. Collectively, curcumin nanoformulation studies in OSCC have been largely confined to in vitro systems or immunodeficient xenograft models; sEV-based delivery in an immunocompetent host has not been previously investigated [15,36].

The present findings reinforce the concept that tumor-derived sEVs actively promote an aggressive tumor phenotype by altering cellular metabolic activity and inducing migratory and invasive activity in recipient cells [8,37]. The inhibitory effects of JCsEV likely reflect not only the intrinsic anti-tumor properties of curcumin, but also improved intracellular delivery efficiency and protection of the compound from premature degradation [13]. Mechanistically, sEV-mediated delivery may enhance curcumin efficacy in vivo through several complementary mechanisms. Encapsulation within the vesicular lipid bilayer may improve the aqueous stability of curcumin, protect it from rapid systemic elimination, and prolong its biological availability under physiological conditions [13,19]. In addition, endogenous vesicle uptake pathways may facilitate intracellular delivery of curcumin to recipient tumor and stromal cells, thereby increasing local drug exposure within the TME [9,12]. These properties may be particularly relevant for curcumin, whose clinical translation is otherwise limited by poor solubility, low bioavailability, and rapid clearance [16]. The observed trend toward a more pronounced therapeutic effect of JCsEV relative to free curcumin, particularly in vivo, therefore aligns with the principle that sEV-mediated delivery can confer pharmacokinetic and cellular delivery advantages, although the direct comparison between JCsEV and free curcumin did not reach statistical significance.

Several limitations should be considered in interpreting these results. First, this was a proof-of-concept study and did not include formal biodistribution, pharmacokinetic, or tissue-targeting analyses of JCsEV in vivo. Second, the immunological consequences of JCsEV treatment were not characterized in detail and the precise influence on TME composition remains to be elucidated. Third, Jurkat cells were used as a standardized vesicle source, which may not fully recapitulate the surface composition or functional repertoire of primary human cell-derived sEVs. Fourth, while PCI-30 cells were used as an OSCC model, the inclusion of additional OSCC-specific cell lines would further strengthen tumor-type specificity. The use of A549 and UMSCC90 cells was intended to capture broader epithelial tumor biology and to assess whether the observed effects are conserved across different epithelial cancer contexts. Additionally, a single dosing regimen and one model cargo were evaluated; the relatively modest sample sizes may have limited the statistical power to detect subtle between-group differences.

## 5. Conclusions

sEV-mediated delivery of curcumin potently suppresses tumor-promoting cellular behavior in vitro and significantly reduces tumor progression in an immunocompetent murine OSCC model. These results establish proof-of-concept for sEVs as functional nanocarriers for anti-tumor drug delivery. Future work should address in vivo biodistribution, immune modulation, and dose optimization to advance this approach toward clinical translation. Overall, sEV-based nanomedicine represents a compelling and therapeutically relevant strategy for the treatment of OSCC.

## Data Availability

The original contributions presented in this study are included in the article/Supplementary Materials. Further inquiries can be directed to the corresponding author.

## Supplementary Materials

The following supporting information can be downloaded at: https://www.mdpi.com/article/10.3390/cancers18101586/s1, Figure S1: Western blotting of sEVs; Figure S2: Macroscopic images of all excised tongues from the 4-NQO murine oral carcinogenesis model.
