# Supplementary figures and images for: Extracellular Vesicle-Mediated Delivery of Curcumin Suppresses Tumor Progression in Murine Oral Squamous Cell Carcinoma
Source: Cancers (Basel). 2026 May 13;18(10):1586. doi: 10.3390/cancers18101586 (PMC13204880; doi:10.3390/cancers18101586)

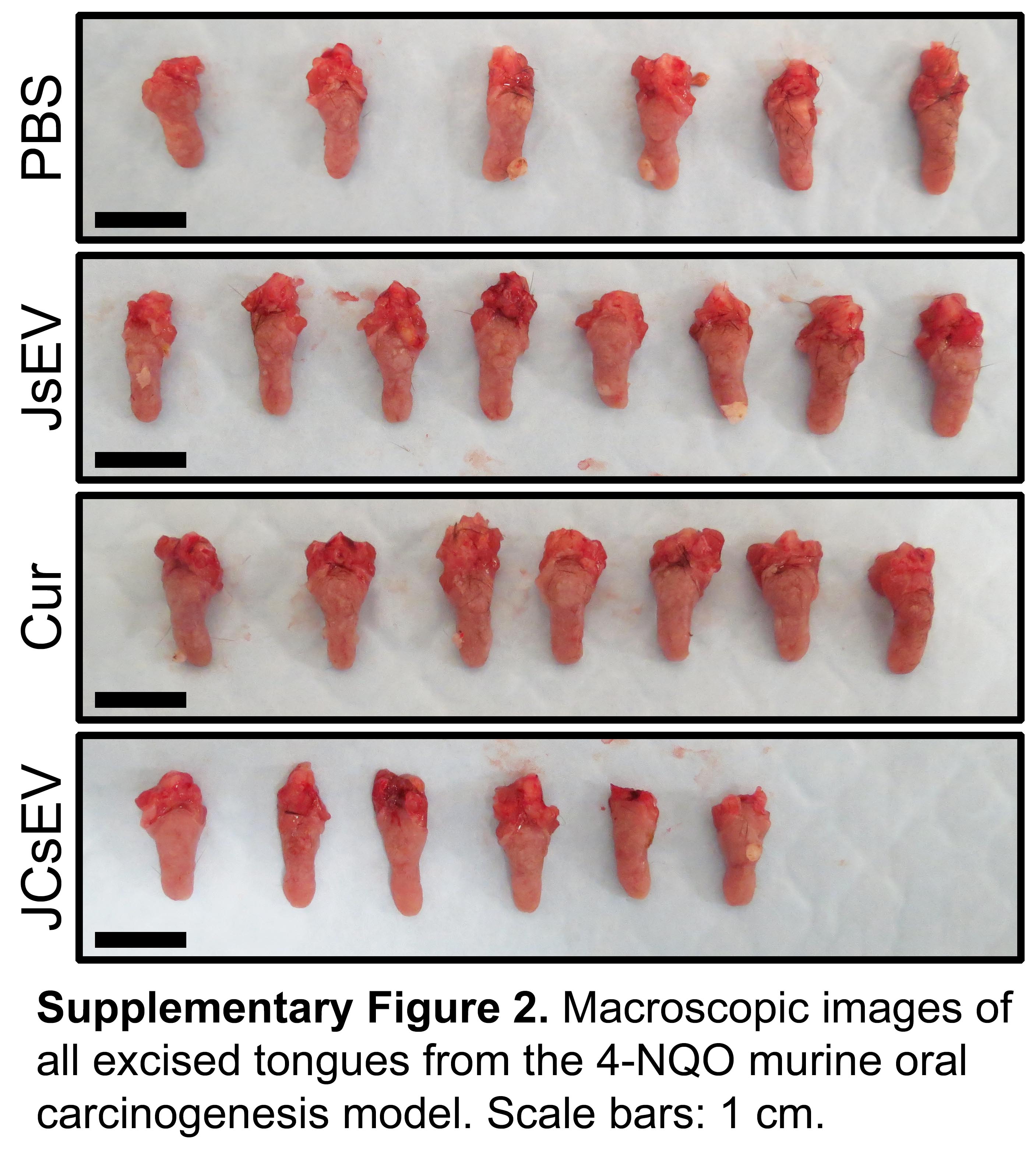

Supplement: Supplementary file 1 [file cancers-18-01586-s001.zip › Supplementary Figure 2.jpg]

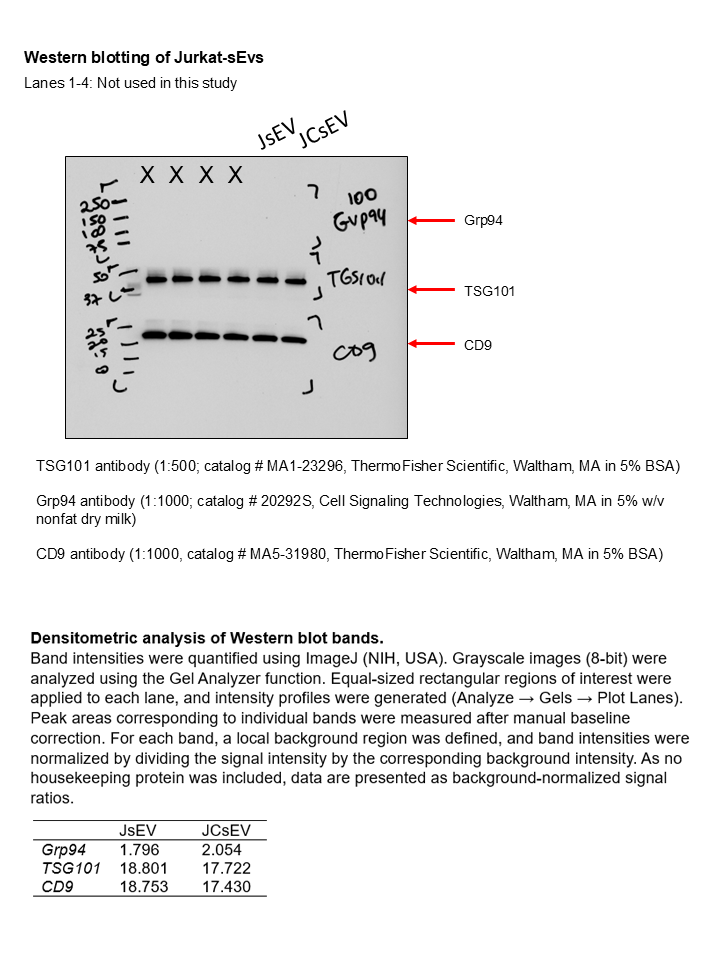

Supplement: Supplementary file 1 [file cancers-18-01586-s001.zip › Supplementary Figure 1 new.tif]
